# O.4.6-3 How collaboration between school, health & sport policy areas resulted in an online platform to activate adolescents: SWEEP

**DOI:** 10.1093/eurpub/ckad133.222

**Published:** 2023-09-11

**Authors:** Femke De Meester

**Affiliations:** Flanders Institute for Healthy Living, Belgium; femke.demeester@gezondleven.be

## Abstract

**Purpose:**

Excessive sedentary behaviour and low levels of physical activity are associated with several adverse physiological and psychological health indicators in adolescents. Yet, many adolescents spend the majority of the day sedentary and adolescents' participation rates in physical activity are low. Therefore, the Flanders Institute for Healthy Living and the Flemish Agency for Care and Health, Sports Flanders, MOEV and the Department of Education and Training combined their forces to develop, evaluate and implement a multi-component intervention (SWEEP) to activate 12 to 18 year old adolescents during school hours and their leisure time.

**Project or policy description:**

The SWEEP intervention is evidence-based and was developed iteratively with adolescents and secondary school teachers using a participatory approach. It is a project for all young people, but with extra attention for those whom we know experience high barriers to exercise (e.g. girls, young people with a migration background, young people in a socially vulnerable situation, older adolescents). SWEEP is an online platform with a section for adolescents and a section for teachers. Both start from a short questionnaire with which adolescents can discover who they are (there are 6 profiles) and teachers can discover how they can get young people into action, how they can upgrade their classes with more movement and which actions suit them best. After they filled in the questionnaire, adolescents are offered activities in line with their profile and therefore with their interests. The teachers get feasible tips, teaching materials and boxes with challenges that can be used in any subject and that matches their own preferences, capabilities and interests.

**Conclusion:**

Between April 2022 and October 2022, SWEEP was pilot tested in the province Antwerp in Flanders. The results of the process- and product evaluation were available in spring of 2023. Overall, SWEEP was generally well accepted and based on the results, the feedback and the suggestions SWEEP was optimized and launched in March 2023 in Flanders.

